# Soluble IL‐2 receptor levels support diagnosis of sarcoidosis‐like reaction in melanoma patients on immunotherapy – a diagnostic algorithm based on a single center retrospective study

**DOI:** 10.1111/ddg.15727

**Published:** 2025-08-31

**Authors:** Philipp Gussek, Julia Mentzel, Maxime Ablefoni, Mirjana Ziemer

**Affiliations:** ^1^ Department of Dermatology Venereology and Allergology University Medical Center Leipzig Leipzig Germany; ^2^ Department of Diagnostic and Interventional Radiology University Medical Center Leipzig Leipzig Germany; ^3^ Department for Interventional Radiology Helios Park‐Klinikum Leipzig Germany

**Keywords:** diagnostic algorithm, immune checkpoint inhibitor therapy, melanoma, sarcoidal reactions, soluble IL‐2‐receptor

## Abstract

**Background and Objectives:**

Drug‐induced sarcoidosis‐like reaction (DISR) is an adverse event with emerging importance during immune checkpoint inhibitor (ICI) treatment in melanoma patients. Its reported frequency varies widely, making accurate diagnosis crucial. Distinguishing DISR from tumor progression is challenging, and misdiagnosis may lead to detrimental treatment changes. Thus, reliable diagnostic markers complementing histopathology as well as a diagnostic algorithm are needed.

**Patients and methods:**

This single‐center retrospective study, conducted at the University Medical Center in Leipzig, Germany, from 09/2019 to 06/2021, assessed DISR prevalence in melanoma patients treated with ICI for metastatic melanoma. We examined clinical characteristics, radiology, histopathology, and serum parameters.

**Results:**

From a total of 177 patients 19 patients were suspicious for DISR. Of those, DISR was diagnosed in seven patients. In a further nine patients DISR was highly probable based on radiological findings and noticeably increased soluble interleukin‐2‐receptor (sIL2R) levels, resulting in DISR prevalence of 9%. No patient required permanent discontinuation of ICI due to DISR.

**Conclusions:**

In melanoma patients receiving ICI, DISR is common. Histological confirmation through mediastinoscopy or pulmonary wedge resection offers the highest diagnostic accuracy. When histology is not available, sIL2R levels can aid diagnosis. We propose an algorithm to distinguish DISR from tumor progression.

## INTRODUCTION

Drug induced sarcoidosis‐like reaction (DISR) is a systemic granulomatous inflammatory reaction due to certain drugs. With their broad application, immune checkpoint inhibitors (ICI) become increasingly important as medicamentous triggers.

In melanoma, DISR occurrence is associated with programmed death‐1 or programmed death‐ligand 1 PD(L)1 inhibitors and cytotoxic T‐lymphocyte‐associated protein 4 (CTLA4) inhibitors or their combinations.^1^, ^2^ The prevalence of DISR in melanoma patients under ICI is variably reported in the literature, and systematic studies with larger patient numbers are still pending. A study examining 147 patients with ICI therapy for advanced melanoma found radiological sarcoid‐like lymphadenopathy in 5% of patients.^3^ In a two‐arm trial of 45 patients receiving blinded nivolumab or combination therapy with ipilimumab and nivolumab, as many as 22% of patients developed DISR, mostly manifesting with bilateral hilar lymphadenopathy.^1^ A retrospective analysis of 457 patients receiving either anti‐PD1 monotherapy or combined therapy with anti‐CTLA4/PD1 reported a prevalence of DISR of 4.1% (3.7% PD1 and 6.3% anti‐CTLA4/PD1).^4^

In addition to lymph nodes, DISR commonly manifests in the lungs and skin.^2^, ^5^ Skin manifestations comprise reddish‐brown macules, papules or plaques, while in the lung interstitial pulmonary infiltrates are typical.^6^ Less commonly, the central nervous system, pituitary gland, spleen, and bones are affected.^5^

The pathogenesis of DISR is not fully understood. As compared to genuine sarcoidosis, DISR is characterized by an increased immune response to an unknown agent, which might include treatment‐released tumor antigens in melanoma.^7^, ^8^, ^9^ T lymphocytes produce increased Th1‐associated cytokines such as interferon‐γ and interleukin 2. Those activate macrophages, which recruit Th17 cells.^10^ Biomarkers, such as serum levels of lung endothelium‐bound angiotensin converting enzyme (ACE) and soluble interleukin‐2‐receptor (sIL2R), have been proposed to facilitate diagnosis, although there is no single specific marker.^11^

Soluble IL2R reflects the activation of the host immune system.^12^ It is elevated in sarcoidosis and correlates with disease activity.^13^, ^14^ It can also be elevated in other inflammatory disorders, especially lung diseases such as idiopathic pulmonary fibrosis or hypersensitivity pneumonitis.^13^ In addition, tumor burden itself may influence sIL2R levels. A longitudinal study examined levels of sIL2R in 242 patients with various stages of melanoma. Soluble IL2R levels were significantly higher than in healthy control subjects. Moreover, higher levels of sIL2R correlated with disease progression and poorer prognosis.^15^ sIL2R levels may normalize after curative surgery for melanoma, supporting the hypothesis that there is a direct relationship to tumor presence.^12^

DISR usually occurs after several months of ICI therapy, varying between 2.8 and 8.7 months.^1^, ^2^, ^5^ DISR is asymptomatic in most patients, so ICI treatment could usually proceed uninterrupted.^1^, ^16^ In some cases, short‐term systemic steroid therapy was necessary.^16^ DISR is usually suspected based on radiological imaging. A typical presentation of DISR/sarcoidosis in computed tomography (CT) and fluorodeoxyglucose 18F positron emission tomography‐CT (FDG PET/CT) is a butterfly‐like, relatively symmetrical (hypermetabolic) mediastinal and bilateral hilar lymphadenopathy.^17^, ^18^ Less commonly, an uptake of (18)F‐FDG in portocaval lymph nodes or subcutaneous hypermetabolic nodules due to noncaseating granulomas has been described.^19^ DISR can mimic disease progression in several constellations, and may thus pose significant challenges to treating physicians.^20^

## PATIENTS, MATERIAL AND METHODS

We retrospectively investigated data of all patients treated in the skin cancer center of the University Medical Center Leipzig who received ICI therapy for stages III–IV melanoma from September 2019 until June 2021 (n = 177). The local ethics committee approved the study (no. 005/22‐ek). We selected and further characterized all patients with suspicious radiological pulmonary, hilar, or mediastinal findings and/or a sarcoidal skin reaction.

Apart from clinical data, we re‐assessed histopathological results of routinely performed biopsies and re‐evaluated the radiological images. In addition, we compared sIL2R and ACE levels as well as tumor parameters. All collected data are summarized in Table S1.

### Evaluation of clinical data

Based on medical charts of the university SAP electronical documentation system we collected patients demographic data, tumor parameters and course of melanoma disease.

### Evaluation of radiological images

Radiological routine tumor staging images were re‐evaluated by an experienced radiologist (M.A.) for suspicious signs of DISR. Radiological findings were grouped into typical peripheral lymph node involvement, i.e., hilar or mediastinal lymphadenopathy, and typical pulmonary involvement, i.e., a fine reticular‐nodular pattern. Further, atypical or nonspecific signs of pulmonary manifestations of DISR were described, such as pulmonary consolidation, ground‐glass opacity, or patterns not otherwise specified.

### Skin biopsy samples

Archived skin biopsy samples from patients with clinically suspected cutaneous sarcoidosis were re‐evaluated by a dermatologist board certified in dermatohistopathology (M.Z.). Biopsy material comprised 4–6 mm punch biopsies, fixed in 4% buffered formalin, embedded in paraffin, and stained with hematoxylin and eosin (HE).

### Invasive thoracic diagnostic procedures

In several patients, histopathologic results of endobronchial ultrasound (EBUS), mediastinoscopy or video‐assisted thoracoscopic (VATS) lobectomy were re‐evaluated.

### Soluble IL2R measurement and analysis

Soluble IL2R levels were evaluated in all 19 patients with suspected DISR. In routine practice, the parameter was collected at the time of CT morphological suspicion of DISR. The normal range for sIL2R was 158–623 U/l. Randomly selected sIL2R levels from 19 of the 177 patients in whom a DISR was not suspected after several months of ICI therapy served as a comparator group. Additionally, two patients with manifest tumor burden and with immune‐related adverse events (irAE) other than DISR were evaluated. Statistical analysis was performed using SPSS version 27.

### Criteria for classification into the probability groups for DISR

The evaluation of the probability of DISR was based on the following criteria: only patients with a histologically confirmed radiological presentation of a sarcoidal reaction – via endobronchial ultrasound (EBUS)‐guided biopsy, mediastinoscopic biopsy, video‐assisted thoracoscopic (VATS) lobectomy, or pulmonary wedge resection – occurring for the first time under ICI therapy were considered to have “definite” DISR. Patients with typical radiological signs without histopathological confirmation but elevated sIL2R/ACE and/or response to systemic steroids were considered “probable” DISR (either no biopsy was taken or the obtained material was too sparse or yielded inconclusive results). Such patients mostly had isolated pulmonary or bilateral hilar lymphadenopathy and otherwise unremarkable tumor parameters.

## RESULTS

Of the n = 177 patients (102 male [57.6%]/75 female [42.4%]) who received ICI therapy, we identified 19 who presented with pulmonary, hilar, and/or mediastinal lymphadenopathy, and/or a sarcoidal skin reaction (mean age 60 years, 14 male) (Table S1). We identified 16 patients (9%) with either *definite* (4%) *or probable* (5%) DISR of which 13 patients were male (81%). Three patients presented sarcoidal changes in baseline staging CT already before starting ICI therapy, and thus were evaluated as “no DISR” (Table S1). Two patients (numbers 10 and 12) were classified as “probable DISR” despite concurrent melanoma metastases, based on the typical radiological presentation, elevated sIL2R/ACE levels, and/or regression of symptoms and lesions under systemic steroid therapy.

At the time of DISR diagnosis seven of the 16 patients were on adjuvant therapy and nine received ICI treatment for advanced disease (palliative). The majority of *definite* DISR were diagnosed in adjuvant‐treated patients (5, compared with 2 in the palliative situation). In advanced stage IV patients the diagnostic certainty decreased, and in most cases only a *probable* DISR was assumed.

In the 16 patients with *definite* and *probable* DISR, clinical or radiological manifestations occurred after a mean time of 272 days (median of 8.9 months [1.2–20.9 months]).

Seven out of 16 DISR patients were symptomatic, with four presenting pulmonary or systemic symptoms like cough and dyspnea and three presenting skin manifestations. In one patient steroid therapy had to be initiated due to cough and dyspnea. In the remaining cases, symptoms were mild and therefore not treated.

With respect to treatment response, three of seven patients with definite DISR showed progressive or relapsed melanoma (2 in the advanced and 1 in the adjuvant setting). Another three patients in the adjuvant situation remained relapse‐free and one in the palliative situation showed complete remission.

### Radiological imaging

In most patients, the radiological imaging performed was CT, and in two patients, PET‐CT. Six out of 16 patients showed a bilateral hilar or mediastinal lymphadenopathy as the only radiological evidence of DISR/sarcoidosis. Furthermore, ten of 16 patients displayed pulmonary involvement (among those were seven cases with both pulmonary involvement and mediastinal or hilar lymphadenopathy). The typical pulmonary picture of sarcoidosis manifestation with a fine reticular‐nodular pattern was found in two patients (2/16). This corresponds to the literature.^17^ In addition, atypical or nonspecific signs of pulmonary manifestations of DISR/sarcoidosis were identified in eight cases (pulmonary consolidation, ground‐glass opacity, or patterns not otherwise specified).

### Skin manifestations

**FIGURE 1:**
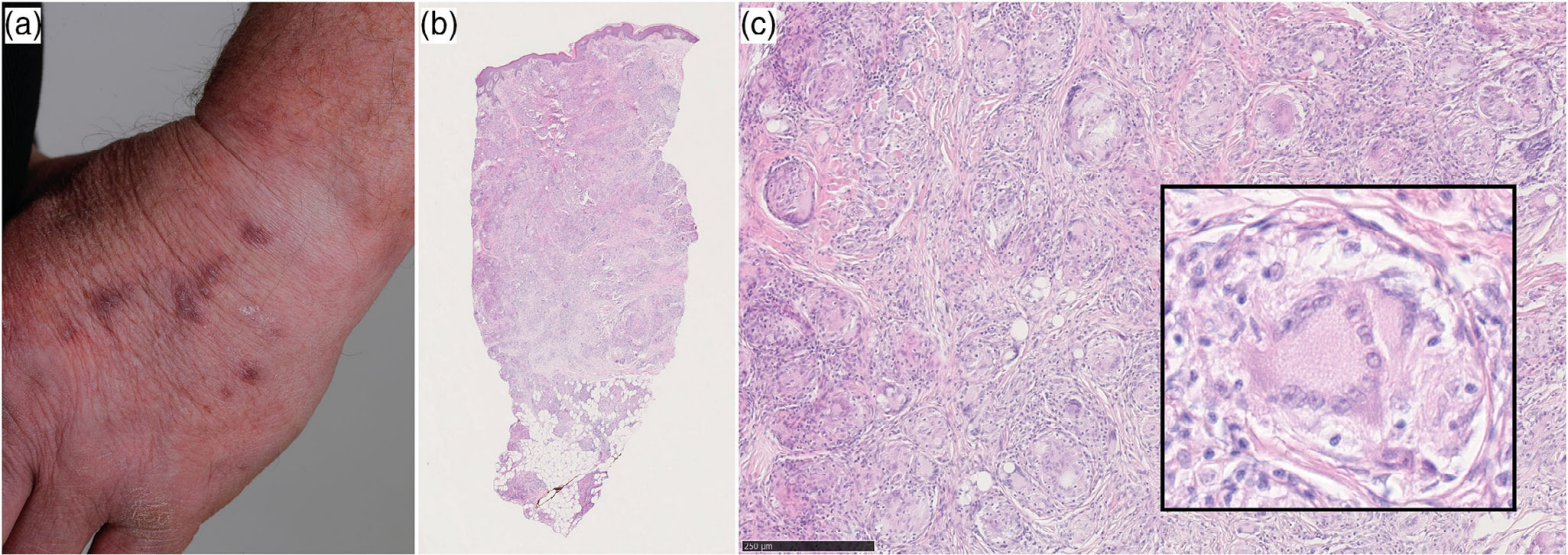
Clinical and histological presentation of a sarcoidal skin reaction in a patient receiving combination therapy with ipilimumab/nivolumab. (a) Reddish‐brown papules and plaques on the dorsum of the hand especially in areas of previous intravenous cannulation (Koebner phenomenon). (b) Histopathologic changes (hematoxylin‐eosin,12,5×) of a skin biopsy from the hand with dermal and subcutaneous granulomatous inflammation. (c) Higher magnification (100×, detail: 400×) shows nodular infiltrates of epithelioid macrophages throughout the dermis with some giant cells (*).

Three patients developed sarcoidal skin reactions, two presenting a concomitant pulmonary and one concomitant pulmonary/hilar and mediastinal involvement. One patient presented with reddish‐brown papules and nodules on both arms under combination therapy with ipilimumab/nivolumab (Figure 1a, Table S1, patient number 5). The lesions appeared also at sites of previous intravenous cannulation, as a Koebner phenomenon. A pandermal and subcutaneous sarcoidal inflammation was confirmed histologically (Figure 1b,c). The second patient was on adjuvant therapy with pembrolizumab and developed a single subcutaneous nodule on the forearm within 6 weeks, also confirmed as sarcoidal granulomatous dermatitis histologically (Table S1, patient number 4). The third patient developed clinical signs of scar sarcoidosis after treatment with adjuvant pembrolizumab (Table S1, patient number 3). He refused a skin biopsy but reported a history of acute sarcoidosis with similar symptoms 14 years earlier.

### Histological confirmation from invasive thoracic diagnostic procedures

Of the 16 patients with *definite* or *probable* DISR, six underwent endobronchial ultrasound guided biopsy, which confirmed sarcoidosis in three cases. The other three cases showed non‐specific inflammatory changes, or the biopsy material was not considered representative. Depending on the anatomical site of the lesions, these patients underwent either a pulmonal VATS or mediastinoscopy. In total, wedge resections of the lung via VATS or mediastinoscopy (one case) were performed in five patients, with sarcoidal inflammation confirmed in 100% of cases.

### Serological markers: ACE and sIL2R

**FIGURE 2:**
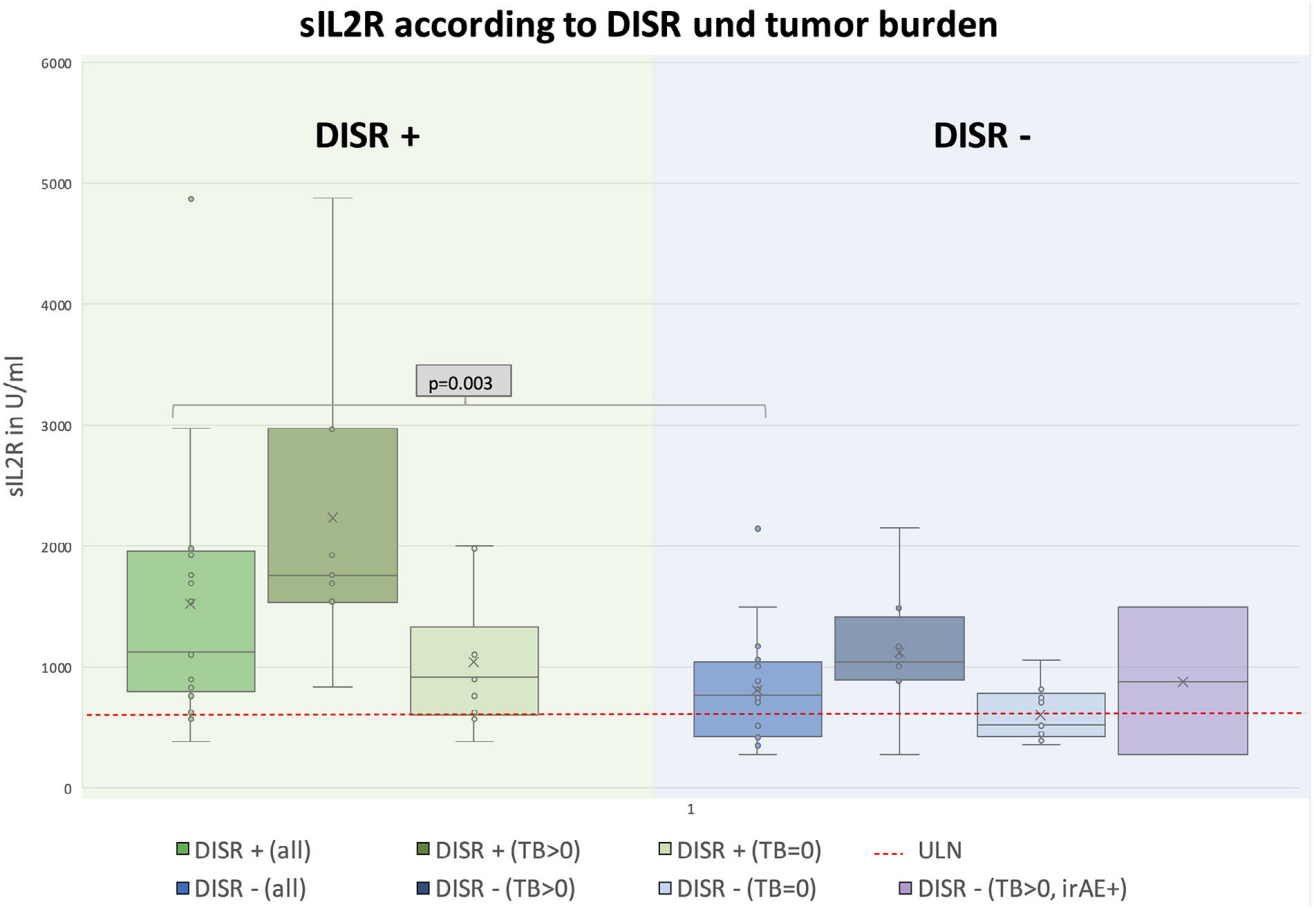
Boxplot showing sIL2R values in a cohort of n = 35 melanoma patients under ICI. n = 16 patients were diagnosed with DISR (“DISR +”), n = 19 did not show signs of DISR (“DISR –“). Groups were further split according to tumor burden, i.e., either tumor burden was > 0 (“TB>0”) or no tumor manifestation was present (TB = 0). The upper level of normal (ULN) was assumed to be 623 U/ml. The “DISR +” group had significantly higher levels of sIL2R than the “DISR –“ group (1,519.4 U/ml (approx. 2.5 x ULN) ± 264.8 vs. 863.1 U/ml ± 113.1, p = 0.003). Further, the TB>0 group demonstrated significantly higher sIL2R values with maximum values in patients with DISR (DISR +/TB>0 group: (2,317.2 U/ml ± 583.4). Lowest values occurred almost exclusively in patients with neither DISR nor tumor burden (572.5 U/ml ± 56.4). Two patients were evaluated with immune‐related adverse events (irAE) unrelated to DISR and TB>0, and only showed slightly elevated sIL2R values.

A two‐factor ANOVA with the between‐subjects factors “tumor burden” (yes/no) and “DISR” (yes/no) revealed significant main effects for both factors (DISR: F [1, 31] = 10.69; p = 0.003; η_p_^2^ = 0.256, and tumor burden: F [1, 31] = 12.83; p < 0.001; η_p_^2^ = 0.325, respectively). Soluble IL2R was elevated in almost all cases with definite and probable DISR (15/16). ACE was elevated in 3 of 7 definite cases and in 2 of 9 probable cases. Patients 8, 10, 14, and 15 had normal values but were receiving ACE inhibitor treatment. We compared sIL2R levels of patients with DISR under ICI (n = 16) with a cohort of 19 control patients under ICI therapy without suspected DISR. Regarding gender and age distribution in the DISR and non‐DISR groups, 13 of 16 and 12 of 19 patients were men, with mean ages of 58.6 and 67.7 years, respectively. Soluble IL2R levels were higher in the DISR vs. non‐DISR group (1519.4 U/ml ± 264.8 vs. 863.1 U/ml ± 113.1). Moreover, sIL2R levels were also higher in the group with tumor burden compared to the group with no tumor burden (1638.5 U/ml ± 285.5 vs. 806.7 U/ml ± 104.1). Highest values of sIL2R were measured in patients with both DISR and tumor burden (2317.2 U/ml ± 583.4) and lowest values in patients with neither DISR nor tumor burden (572.5 U/ml ± 56.4) (Figure 2). In sum, sIL2R values greater than 2.5 times the ULN almost only occurred in the patient groups with DISR. In contrast, normal sIL2R values were found almost exclusively in groups without DISR.

### A working algorithm for diagnosis of DISR

**FIGURE 3:**
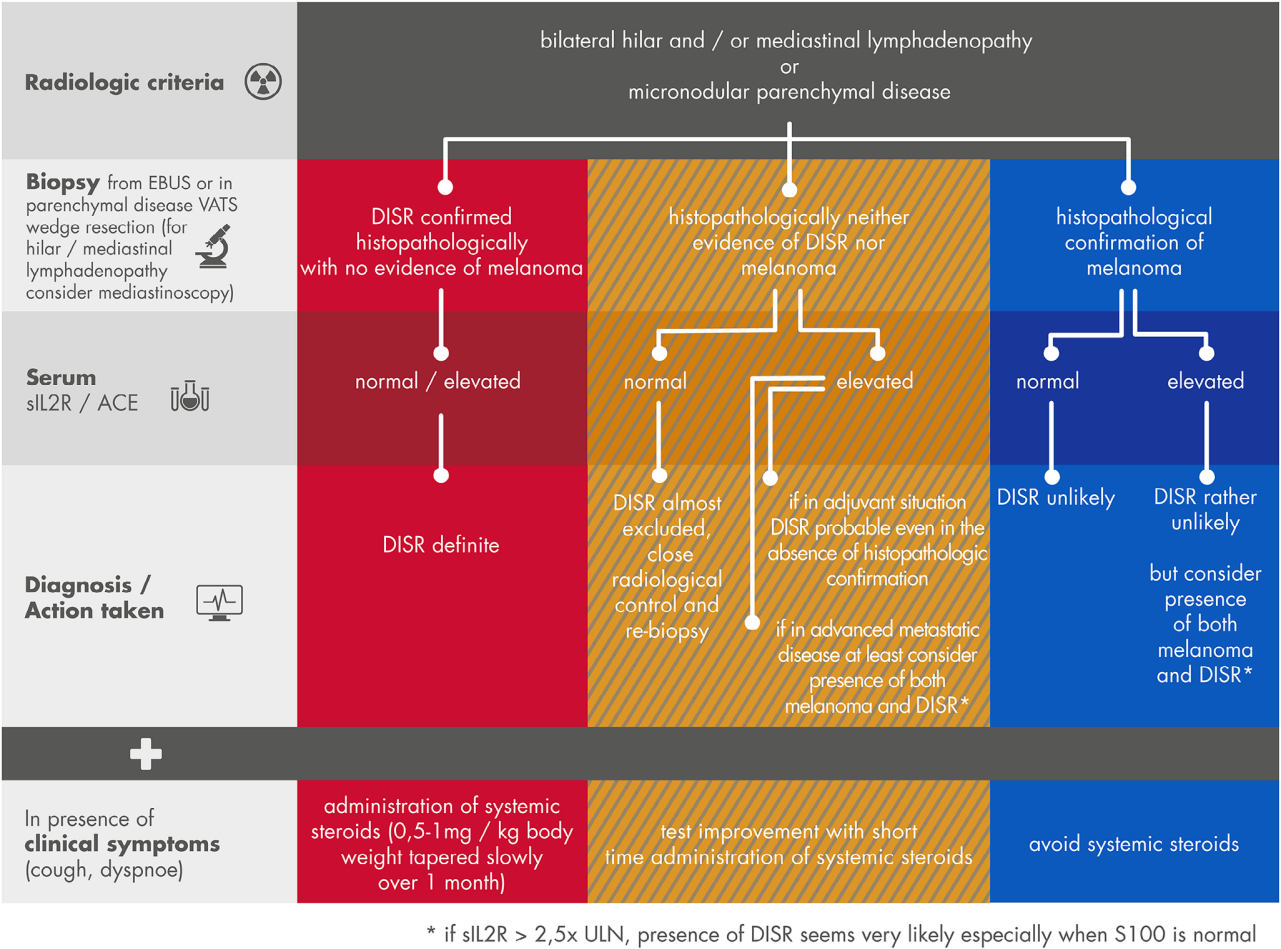
Diagnostic algorithm for differential diagnosis of DISR versus melanoma/tumor metastasis. Radiological, histological, and serological findings as well as clinical symptoms and response to steroid therapy should be taken into account when evaluating DISR. Short‐term administration of corticosteroids refers to 14 days or less with 0.5–1mg/kg bodyweight prednisolone equivalent. Although this is the largest cohort to date in which the occurrence of DISR under ICI has been investigated, the number of patients remains relatively small. Our recommendations and conclusions, in particular the diagnostic algorithm involving sIL2R, should therefore be applied with caution on a case‐by‐case basis.

Based on radiological, histopathological, serological and clinical parameters we suggest a working algorithm to facilitate diagnosis. It may help evaluating the likelihood of DISR and differentiation from melanoma metastasis or disease progression in clinical practice (Figure 3).

## DISCUSSION

### Prevalence of DISR

In this study, we retrospectively analyzed 177 patients with metastatic melanoma undergoing adjuvant or palliative ICI and diagnosed *definite or probable* DISR in 9% of patients.

Occurrence of DISR in large melanoma trials ranged from 1.4% to 5.8%^21^, ^22^, ^23^, ^24^ or was not reported.^25^ One of the largest comparable studies to date on DISR by Chorti et al. with 45 adjuvant melanoma patients receiving combined ICI with ipilimumab and nivolumab even reported a prevalence of 22%.^1^ Chorti et al. discussed whether DISR was more common in the adjuvant setting than in the palliative setting, as they found a much higher rate of DISR in their adjuvant cohort compared with the literature. Another possible explanation for the prevalence might be the combination of ipilimumab and nivolumab.

In the DISR patient group we studied, no clear difference in the prevalence of DISR was observed between patients receiving ICI in the adjuvant setting or for advanced disease, or between combination and single‐agent therapy. Overall, one‐fifth of patients in our cohort received combination therapy with ipilimumab and nivolumab, and they were equally distributed in all DISR probability groups. Although our cohort is relatively small and the significance therefore remains limited, this does suggest that the impact of combination therapy is rather small compared to monotherapy.

It was noticeable that patients who had *definite* DISR in our group were exclusively men. Even in the cases assessed as *probable*, only three of nine were female. Similarly, all patients identified by Dimitriou et al. as having DISR were male. This would be consistent with the androtropism known for “classic” sarcoidosis.^26^ However, some studies also found no sex difference^2^ or only a mild gynecotropism.^1^ Similar to previously described cases,^1^, ^2^ only a subset of patients in our cohort were symptomatic (7 of 16).

A history of sarcoidosis may predispose patients to developing DISR. In case series described in the literature, preexisting sarcoidosis is very rare. For example, a review of 55 cases of sarcoid‐like granulomas in cancer patients under ICI treatment reported a medical history of sarcoidosis in only one patient.^5^ In another series of 32 patients under ICI with a history of sarcoidosis only one patient had an aggravation of symptoms.^27^ In our cohort, one patient with DISR had a history of acute sarcoidosis diagnosed fifteen years earlier. Because of the non‐symptomatic course of DISR, immunotherapy was not interrupted, and no steroid therapy was required in this patient.

That DISR and irAEs in general might be more common with ICI therapy than suspected was suggested by an autopsy study. Here, post‐mortem examinations were performed in a patient with advanced disseminated melanoma. A sarcoidal reaction of the lung was found, which was clinically and radiologically interpreted as immune related pneumonitis while the patient was alive. In addition, a systemic inflammatory reaction was found in several other organ systems, which was had not been clinically apparent before.^28^

Finally, it should be mentioned again that melanoma disease itself appears to be a predisposing factor for sarcoidal reactions.^29^

### Diagnostic tools

Our data confirm that radiological imaging is the most important tool for detecting DISR, as all 16 cases in our cohort exhibited predominantly typical radiological findings on (PET‐)CT scans. As in the literature and in our patient population, DISR first appeared as bilateral hilar, mediastinal, and/or pulmonary lymphadenopathy on imaging in tumor staging. Moreover, skin manifestations occurred in three out of 16 patients with DISR. All patients with cutaneous involvement of DISR also showed concurrent pulmonary or mediastinal involvement, with skin manifestations either preceding the radiologic findings or aiding in their classification as DISR. Considering that a considerable percentage of patients with DISR had cutaneous manifestations, treating physicians should perform a thorough physical skin examination when DISR is suspected. Neither computed tomography nor PET‐CT alone can reliably differentiate between tumor manifestation and DISR. DISR as well as tumor metastasis are positive in FDG‐PET, especially for lymph nodes.^19^ A characteristic distribution for DISR such as the “butterfly pattern” can provide an important clue.^18^ In seven patients from our cohort, either radiological (n = 6) or clinical suspicion (n = 1) of DISR could be confirmed histologically or cytologically. Histological confirmation was most often achieved via mediastinoscopic biopsy or wedge resection (100%) and considerably less frequently by transbronchial biopsy (50%). In many cases, the amount of tissue obtained by transbronchial biopsy was insufficient for a definite diagnosis. Moreover, a sarcoidal reaction and a metastasis might be present at the same time, an aspect which can be more easily missed in a fine needle biopsy.^30^ Some authors have speculated that a sarcoidal reaction may be a sign of an (effective) immune reaction against tumor cells, since across tumor entities metastatic disease is more rare and survival outcomes are better in patients with confirmed non‐infectious granulomas.^31^, ^32^

Nevertheless, apart from histopathology, reliable markers for the diagnosis of DISR are lacking. Serologic parameters may help clarify cases before deciding on invasive investigations or when a biopsy for histopathologic confirmation is difficult to obtain. We therefore investigated sIL2R levels in melanoma patients undergoing ICI therapy with and without DISR. Serum IL2R levels in patients with suspected DISR on radiological imaging in adjuvant treatment settings (where no tumor manifestations are assumed to be present) were the most reliable. Thus, patients with hilar or mediastinal lymphadenopathy and considerably increased sIL2R levels but otherwise normal imaging results and normal S100 are most likely to have DISR. Those patients showed significantly higher sIL2R levels than patients without DISR in our patient cohort. Even a small increase in sIL2R levels correlates with the likelihood of DISR. Since only 2 of 16 patients with either *definite* or *probable* DISR showed no elevation of sIL2R levels, it would appear that sIL2R has a high sensitivity and negative predictive value for the diagnosis of DISR (87.5% and 77.8%, respectively), and may therefore be considered a useful diagnostic tool. Nevertheless, patients with tumor burden and without DISR also had increased sIL2R, although these levels were comparatively lower. This is consistent with results from previous studies showing that sIL2R levels are elevated in patients with melanoma due to activation of the host immune system and correlate with cancer progression.^33^ Therefore, for DISR assessment, tumor burden must be considered as a confounding factor in the interpretation of sIL2R values. Especially in patients with advanced melanoma disease, the distinction of DISR and tumor progression is problematic. Since sIL2R also correlates with disease activity in sarcoidosis a cumulative effect on sIL2R levels in patients with high tumor burden and DISR is plausible and consistent with our findings.^34^, ^35^ Our data showed a trend (not statistically significant) toward the highest sIL2R levels in patients with both DISR and melanoma progression (approximately 3.5 times the upper limit of normal [ULN]). For this reason, serum sIL2R concentrations in patients with advanced melanoma are unlikely to help distinguish DISR from further tumor progression, unless serum S100 is low and has previously correlated with melanoma activity in the individual patient. In these patients, histopathological confirmation of the diagnosis is particularly important. On the other hand, in the adjuvant situation (when tumor burden is 0) or when diagnostic imaging and tumor marker S100 indicate stable disease in the presence of tumor burden, our data suggest that sIL2R values greater than 2.5 times the ULN are more likely to indicate DISR, as these high values were almost only measured in the presence of DISR. Another confounding factor for the evaluation of DISR may be the presence of other irAEs since they can also cause an increase in sIL2R levels.^36^ To illustrate the issue, we have shown elevated sIL2R levels in two patients with simultaneous presence of tumor burden and of irAEs other than DISR. To investigate the diagnostic validity of sILR2 for DISR, future studies with larger cohorts of patients with irAEs with and without tumor burden are necessary. Until then, treating physicians should exclude other irAEs when evaluating sIL2R for the presence of DISR. In our cohort, one of the 16 patients with DISR also presented with other irAEs (fatigue and myalgia) at the onset of DISR.

In summary, our data indicate that, especially in the adjuvant treatment setting with inconclusive results from imaging and instrumental diagnostics, high levels of sIL2R (> 2.5 x ULN) are more indicative of DISR than of tumor relapse, whereas normal levels of sIL2R make DISR very unlikely.

Since the validation of the diagnostic value of serological markers requires specifically designed studies with hundreds to thousands of subjects, it must be emphasized that our cut‐off values represent only an initial estimate based on a small cohort (n = 16).

ACE activity in serum may be a less reliable marker, as it tends to be higher in heavy smokers and lower in patients receiving ACE inhibitors – factors that together affect a large proportion of patients.^37^ However, ACE activity is only slightly elevated in smokers and does not usually exceed the ULN. Nevertheless, since patients 8, 10, 14, and 15 in our study were receiving ACE inhibitors, their ACE levels may have been underestimated.

### Treatment response

It also remains unclear whether the occurrence of DISR correlates with a better treatment response. While a weak correlation has been described between the severity of irAEs and treatment response,^38^ no such evaluation is currently available for DISR. Chorti et al. studied a cohort of patients receiving ICI in the adjuvant setting and saw no difference in relapse rate between patients with and without DISR. However, they discussed whether there might be a benefit for patients in palliative care ‐ an aspect that has already been described in the literature.^1^, ^39^, ^40^ We saw no benefit in treatment response in patients with DISR. The response was heterogeneous in both adjuvant and advanced disease settings. In a literature review, 38 of 91 patients with a sarcoidal reaction discontinued therapy. However, changes in therapy did not influence the course of the sarcoidal reaction. In half of the cases, DISR required treatment.^2^ In our cohort, no patient discontinued ICI therapy due to DISR, and only two patients required symptomatic treatment.

### Conclusions: individual approach and working algorithm

Considering all aspects discussed, an individualized approach is essential when DISR is suspected. Clinical features, radiologic criteria, histology, serological parameters, and response to systemic steroid therapy are key diagnostic components for the final diagnosis of DISR (Figure 3).

Histological confirmation remains the gold standard for diagnosis. In individual cases, however, a diagnosis can be made without biopsy if other findings are typical. Our analysis of sIL2R suggests that normal levels virtually exclude DISR. Furthermore, our results suggest that in the adjuvant treatment setting, marked elevations of sIL2R (> 2.5 × ULN) are more indicative of DISR than of tumor relapse – especially when S100 levels and imaging findings are otherwise unremarkable, except for DISR‐like pulmonary and/or hilar/mediastinal changes.

In any case, the awareness of treating physicians should be improved regarding the differential diagnosis of DISR under ICI therapy. To this end, our flowchart with a diagnostic algorithm, along with the subsequent key points, is intended as a practical aid for the treating clinician. The aim is to avoid premature and uncritical interpretation of clinical findings of possible DISR as tumor progression, so as not to deprive patients of potentially valuable therapeutic options.

Although this is the largest cohort under ICI therapy to date in which the occurrence of DISR has been investigated, the number of DISR cases remains relatively small at n = 16. Therefore, our recommendations and conclusions – particularly regarding the diagnostic algorithm and the use of sIL2R as a serological parameter – should be interpreted with appropriate caution in each individual case.

## CONFLICT OF INTEREST STATEMENT

M.Z. has received lecture fees or financial support for congress participation from Bristol‐Myers Squibb, Novartis, AstraZeneca, Pierre Fabre, Sanofi‐Aventis, and Sunpharma. J.M., P.G., and M.A. declare no conflicts of interest.

## Supporting information



Supporting Information
