# Supplementary material for: Soluble IL‐2 receptor levels support diagnosis of sarcoidosis‐like reaction in melanoma patients on immunotherapy – a diagnostic algorithm based on a single center retrospective study
Source: J Dtsch Dermatol Ges. 2025 Aug 31;23(11):1404–12. doi: 10.1111/ddg.15727 (PMC12619049; doi:10.1111/ddg.15727)
Supplement: Supplementary file 1 — Supporting Information [file DDG-23-1404-s001.docx]

Table 1

| Patient no. | Stage (AJCC v.8)* | Sex/age^a^ | ICI^b^ | BRAF^c^ | Symptoms^d^ | Manifestations of sarcoidosis/DISR^e^ | Time to onset of sarcoidosis/DISR^o^ | BAL/EBUS^f^ | Histology^g^ | Stop ICI | ACE/IL2R^i^ | S100 | Steroids^j^ | Course of sarcoidosis^k^ | Response to ICI^l^ | Particularities | Evaluation of DISR |
| --- | --- | --- | --- | --- | --- | --- | --- | --- | --- | --- | --- | --- | --- | --- | --- | --- | --- |
| 1 | IIIB | M/52 | 10aNIVO | WT | None | hil/med | 148 d | Lymphocytosis/sarcoidosis | Transbronchial biopsy: sarcoidosis | No^q^ | ++/++ | N | No | Partially regressed in CT; symptoms regressed under steroids | RFS 36 months | Anthracosis of hilar LN | Definite |
| 2 | IIIB | M/56 | 7aPEMBRO | WT | Dry cough (mild) | Pulm | 183 d | N.p./n.p. | Pulm. wedge resection: sarcoidosis, metastasis and anthracosis | No^r^ | N/+ | N | No | No recurrence after resection | PD (relapse after 11 months) | Coincidence of sarcoidosis, pulmonal anthracosis and pulmonal metastasis | Definite |
| 3 | IIIC | M/53 | 4aPEMBRO | V600E | Scar sarcoidosis | Pulm/hil/med/cut | 90 d | N.p./sarcoidosis | N.p. | No | N/+ | N | No | Partially regressed in PET-CT | RFS 24 months | State after acute sarcoidosis (2005), brother: sarcoidosis | Definite |
| 4 | IV | M/64 | 5aPEMBRO | p.G466V | Subcutaneous nodule forearm | Pulm/cut | 184 d | N.p./n.p. | Skin biopsy: sarcoidosis | No | N/+ | + | No | Completely regressed within 7 months (cut and pulm) | RFS 14 months | Coal stove for 33 years | Definite |
| 5 | IV | M/63 | 3pATEZO, 2pNIVO, 2pIPI/NIVO | V600E | Erythematous nodules and plaques forearm | Pulm/cut | 350 d | N.p./n.p. | Pulm. wedge resection: sarcoidosis | No | ++/++ | + | No | Constant for 11 months | PD | – | Definite |
| 6 | IV | M/52 | 7aNIVO/16pNiVO | WT | None | Med/Pulm | 236 d | N.p./sarcoidosis | Mediastinoscopy + pulm. wedge resection: sarcoidosis and anthracosis | No^s^ | N/+ | N | No | Partially regressed in CT | CR  26 months | Pulmonal anthracosis | Definite |
| 7 | IV | M/64 | 66pNivo | WT | None | Pulm | 635 d | N.p./n.p. | Pulm. wedge resection: sarcoidosis | No^t^ | N/n | N | No | No recurrence after resection | PD | Smoker; coal stove in childhood | Definite |
| 8 | IIIC | M/36 | 2aPEMBRO | V600R | None | Pulm/hil/med | 43 d | N.p./n.p. | N.p. | No | N/++ | N | No | Further progressed in PET-CT | RFS 10 months | No histologic confirmation^m^ | Probable |
| 9 | IIIC | F/78 | 3aNIVO | V600E | None | Pulm/hil/med | 89 d | N.p./n.p. | N.p. | No | N/+ | N | No | UNK | UNK | No histologic confirmation^m^; loss of follow-up | Probable |
| 10 | IV | M/50 | 3pIPI/NIVO | WT | None | Hil/med | 147 d | N.p./n.p. | N.p. | No | N/++ | N | No | Death 3 months later | PD | No histologic confirmation | Probable |
| 11 | IV | M/47 | 3pPEMBRO | p.K601E | Productive cough  (mild) | hil/med | 77 d | N.p./n.p. | N.p. | No | +/+ | N | No | Completely regressed within 8 months | SD | No histologic confirmation | Probable |
| 12 | IV | M/49 | 2pIPI/NIVO, 2pPEMBRO | V600E | Dyspnea, fatigue | Pulm/hil/med | 834 d | No evidence of sarcoidosis/lymphatic hyperplasia | Pulm. wedge resection: metastasis melanoma, no evidence of sarcoidosis | No | ++/++ | N | 50mg tapered  within 14d | Dyspnea, fatigue, pul and hil. lesions regressed under steroids | PD | No histologic confirmation^n^ | Probable |
| 13 | IIIC | F/79 | 7pNIVO | WT | None | Hil/med | 190 d | N.p./lymphatic hyperplasia | N.p. | No | N/n | N | No | Constant med and hil. lymphadenopathy | CR | No histologic confirmation; coal stove > 20 years | Probable |
| 14 | IIIC | M/53 | 17pNIVO | V600E | Cough, dyspnea (mild) | Pulm/med | 671 d | Diminished CD4/CD8 ratio/n.p. | N.p. | No | N/++ | N | No | Completely regressed within 1 year | CR | no histologic confirmation; coal stove for 1 year | Probable |
| 15 | IV | F/82 | 2pPEMBRO | WT | None | Pulm/hil | 35 d | N.p./n.p. | N.p. | No | N/+ | N | No | Constant since 16 months | CR | No histologic confirmation; coal stove for 60 years | Probable |
| 16 | IV | M/60 | 30pNIVO, 3pIPI/NIVO | WT | None | Hil/med | 433 d | N.p./ lymphatic hyperplasia and anthracosis | N.p. | No | N/++ | N | No | Partially regressed in CT within 6 months | PD | No histologic confirmation^o^ | Probable |
| 17 | IIIC | F/77 | pNIVO | WT | None | Med | –35 d | N.p./n.p. | N.p. | No | N/n | N | No | Constant med lymphadenopathy | SD | No histologic confirmation | No DISR |
| 18 | IIID | F/69 | aNIVO | V600K | None | Pulm/hil/med | –30 d | N.p./lymphatic hyperplasia | N.p. | No | N/+ | N | No | UNK | RFS 4 months | No histologic confirmation | No DISR |
| 19 | IV | M/69 | pPEMBRO | WT | None | Pulm | –40 d | N.p./n.p. | Pulm. wedge resection: metastasis melanoma and chronic lymphofollicular inflammation | No | N/++ | N | No | Progressive pulmonal nodules | PD | Acute sarcoidosis 20 yearsa ago | No DISR |

Patients 17–19 were assessed as “no DISR” as radiological signs with pulmonary, hilar, and/or mediastinal lymphadenopathy were present already before ICI

*Melanoma stage at start of immune checkpoint inhibitor (ICI) therapy according to AJCC version 8

^a^Female (F) or male (M)

^b^Type of ICI therapy: adjuvant (a) or treatment in advanced disease (palliative, p) including number of administered cycles (number) of Nivolumab (NIVO, according to a dose of 240 mg, Q2W), Ipilimumab/Nivolumab (3 mg/kg/1 mg/kg, Q3W) Atezolizumab (ATEZO, 1,200 mg, Q3W), Pembrolizumab (PEMBRO, 200 mg, Q3W) until the onset of DISR

^c^BRAF V600 and non-V600 point mutations or wild type (WT)

^d^Symptoms suggestive of sarcoidosis

^e^Sarcoidosis manifestation: pulmonary (pulm), hilar (hil), mediastinal (med), cutaneous or subcutaneous (cut)

^f^Results of bronchoalveolar lavage (BAL) and of endobronchial ultrasound (EBUS), or not performed (n.p.)

^g^Histopathology results after biopsy/resection with the respective technique or if not performed (n.p).

^i^Angiotensin converting enzyme (ACE), soluble interleukin-2-receptor (sIL2R) and S100 in sera as normal (n), elevated (+), considerably elevated (++; i.e. > 2 x ULN) at the time of suspicion of DISR

^j^Indicated as prednisolone dosing equivalence

^k^Course of DISR/sarcoidosis in radiologic imaging/clinically

^l^Treatment response of melanoma: complete remission (CR), stable disease (SD), progressive disease (PD), in adjuvant situation relapse-free survival (RFS) time

^m^Despite no histologic confirmation of sarcoidosis (patient rejected biopsy), certainty of diagnosis of DISR is very high due to typical pattern in PET-CT/CT, very high sIL2R and normal values of S100

^n^Despite no histologic confirmation of sarcoidosis, coincidence of sarcoidosis and pulmonary metastasis is very likely because pulmonary micronodules and bihilar lymphadenopathy regressed after steroid therapy while mediastinal lymph nodes masses enlarged enormously

^o^Despite no histologic confirmation of sarcoidosis, coincidence of hilar sarcoidosis and pulmonary metastasis is very likely because bihilar lymphadenopathy regressed while pulmonary metastasis remained constant

^q^ICI was stopped due to the suspicion of other immune related adverse events, no ICI was necessary in the following

^r^ICI was stopped due to R0-resection at that point, pembrolizumab was not continued due to progress under pembrolizumab, no ICI was necessary in the following

^s^ICI was stopped due to suspicion of progress and not continued

^t^ICI was stopped due to the suspicion of other immune related events and re-induced

*Other abbr.:* n.p., not performed; UNK, unknown
